# A comprehensive review on miR-153: Mechanistic and controversial roles of miR-153 in tumorigenicity of cancer cells

**DOI:** 10.3389/fonc.2022.985897

**Published:** 2022-09-09

**Authors:** Saghar Yousefnia

**Affiliations:** Department of Cell and Molecular Biology and Microbiology, Faculty of Biological Science and Technology, University of Isfahan, Isfahan, Iran

**Keywords:** miR-153, tumorigenicity, non-coding RNAs, metastasis, chemo/radiotherapy resistance

## Abstract

miRNAs play a crucial role in regulating genes involved in cancer progression. Recently, miR-153 has been mainly well-known as a tumor suppressive miRNA modulating genes in proliferation, metastasis, EMT, angiogenesis and drug resistance ability of a variety types of cancer. Mechanistic activity of miR-153 in tumorigenicity has not been fully reviewed. This manuscript presents a comprehensive review on the tumor suppressive activity of miR-153 as well as introducing the controversial role of miR-153 as an oncogenic miRNA in cancer. Furthermore, it summarizes all potential non-coding RNAs such as long non-coding RNAs (LncRNAs), transcribed ultra-conserved regions (T-UCRs) and circular RNAs (CircRNAs) targeting and sponging miR-153. Understanding the critical role of miR-153 in cell growth, metastasis, angiogenesis and drug resistance ability of cancer cells, suggests miR-153 as a potential prognostic biomarker for detecting cancer as well as providing a novel treatment strategy to combat with several types of cancer.

## Introduction

Cancer is one of the most critical diseases with an increasing prevalence rate and high mortality. Cancer is a type of cell transformation and loss of cell differentiation with uncontrolled cell growth. It is also a genetic and epigenetic disorder, having much heterogeneity in both tissue and cellular levels. It is characterized by different properties such as proliferation, migration and invasion, metastasis, angiogenesis and chemo/radio resistance abilities. There are many oncogenes, tumor suppressor and regulatory genes which modulate cancer characterizations through various mechanistic pathways (1, 2).

miRNAs are non-coding small RNAs (∼20–24 nucleotides) which regulate target genes negatively, by binding to complementary sites of 3´ UTR in a specific gene’s mRNA, resulting in mRNA degradation or translation inhibition based on complete or partial complementary, respectively (3). Recently, the role of microRNAs (miRNAs) as oncogenes or tumor suppressor genes, has been confirmed in regulating genes involved in the progression of various types of malignancies. The expression level of numerous miRNAs has modified in different types of cancer cells (4–6). Dysregulation of miRNAs is associated with the development and progression of cancer. Therefore, miRNAs have been proposed for using as prognostic biomarkers, predicting and detecting cancer as well as usage as novel strategy for treatment of cancer (7). miR-153, one of the miRNAs involved in cancer progression and development, has recently been reported in several studies. The two miRNAs genes, miR-153-1 (miR-153-5p) and miR-153-2 (miR-153-3p) with a conserved sequence are evolutionarily-conserved genes in Eukaryotes, Vertebrata, Mammalia, Primates, Hominidae, and Homo located on chromosome 2q35 (Chromosome 2 - NC_000002.12) with 90 bp (https://www.ncbi.nlm.nih.gov/gene/406944) and chromosome 7q36.3 (Chromosome 7-NC_000007.14) with 87 bp (https://ncbi.nlm.nih.gov/gene/406945) Evaluation of genomic sequence of miRNA-153-1 and miRNA-153-2 has been revealed that these miRNAs as intragenic miRNAs, are embedded within the 19th intron of *IA-2* (also known as *PTPRN*) and *IA-2β* (also known as *PTPRN2*), the conserved host genes, respectively (8).

Recently, dysregulation of miR-153 has been verified in different types of cancer cells such as cervical, lung, neuroblastoma and gastric cancer (9–12). It is implicated in cancer pathophysiological processes, including proliferation, apoptosis, invasion, EMT and metastasis, angiogenesis and chemo/radiotherapy resistance (13). Most importantly, miR-153 acts as a tumor suppressive miRNA to reduce stemness phenotypes of cancer cells (14, 15). Both 5′-arm and 3′-arm of the miR-153 precursor suppress tumor initiation and progression through modulating expression of their target genes (16). Besides anti-tumorigenicity activity of miR-153, several studies have reported the oncogenic role of miR-153 in some types of cancer cells (16–18). It recommends a controversial role of miR-153 as both a tumor suppressor gene and an oncogene.

Critical role of miR-153 in different types of cancers and especially the controversial role of miR-153 in tumorigenicity of cancer cells made the author put forth this review, which is the first comprehensive review with details about miR-153, its transcriptional regulation and mechanistic effects on cancer. This review presents a study on the mechanistic roles of miR-153 as either a tumor suppressor gene or an oncogene in proliferation, apoptosis, migration and invasion, metastasis, angiogenesis and chemo/radio resistance ability of cancer cells. Moreover, it introduces a number of non-coding RNAs (ncRNAs) which regulate the expression of miR-153 in different mechanistic pathways of malignancy. Furthermore, it summarizes some suggested strategies for miR-153-related therapy as well as suggesting as a prognostic biomarker that is associated with some pathological parameters. Recognition and understanding the molecular mechanisms modulated by miR-153 in cancer cells, can be effective for discovering novel treatment strategies as well as usage as a specific prognostic biomarker for the prognosis of different types of cancer.

## Transcriptional regulation of miR-153

Primarily, transcriptional regulation of miRNAs is dependent on genomic stability, activity of transcription factors, epigenetic regulation and miRNA biogenesis pathway. There are some potential mechanisms that deregulate miRNAs in cancers including the amplification or deletion of miRNA genes, aberrant activity of transcription factors, epigenetic dysregulation and defects in the miRNA biogenesis pathway (19). miRNAs is regulated by several transcription activators, co-activators or suppressors, so dysregulation of some key transcription factors results in abnormal expression of miRNAs in tumors. Search to discover the potential transcription factors for transcriptional regulation of miR-153 in TRANSFAC database has exhibited the predicted binding sites for transcription factors such as CREB-binding protein (CBP)/p300, cAMP-response element binding protein (CREB), C/EBPß, and ATF4 at promoter sequence of miR-153 (20). Both CBP and p300 interact with CREB/ATF4 in a pathway dependent on the cAMP-dependent protein kinase A and may perform an important role in alteration in gene expression and the regulation of signaling pathways (21). Furthermore, it has been reported that KLF4 may also act as a transcription factor of miR-153 (22). Therefore, the regulation of miR-153 may be mediated by a group of transcription factors and dysregulation of these transcription factors may result in abnormal expression of miR-153 in tumors. Also, it has been reported that the intragenic cryptic promoter of miR-153 may be regulated epigenetically with a site of enrichment for H3K4me3 in hippocampal neurons (20). There is no more evidence on transcriptional regulation of miR-153 in cancer. Nevertheless, further studies are needed to discover the exact mechanism of miR-153 dysregulation in cancer.

miRNA-153 is transcribed by RNA polymerase II as a primary transcripts (pri-miRNAs). The primary transcript is cleaved by the Drosha ribonuclease III enzyme to produce an approximately 70-nt stem-loop precursor miRNA (pre-miRNA), which is further cleaved by the cytoplasmic Dicer ribonuclease to generate the mature miRNA-153 (https://ncbi.nlm.nih.gov/gene/406945). The mature miRNA-153 is incorporated into a RNA-induced silencing complex (RISC), which recognizes target mRNAs through base pairing with the miRNA and results in translational inhibition or destabilization of the target mRNAs (8).

## Role of miR-153 in proliferation of cancer cells

**Figure 1:**
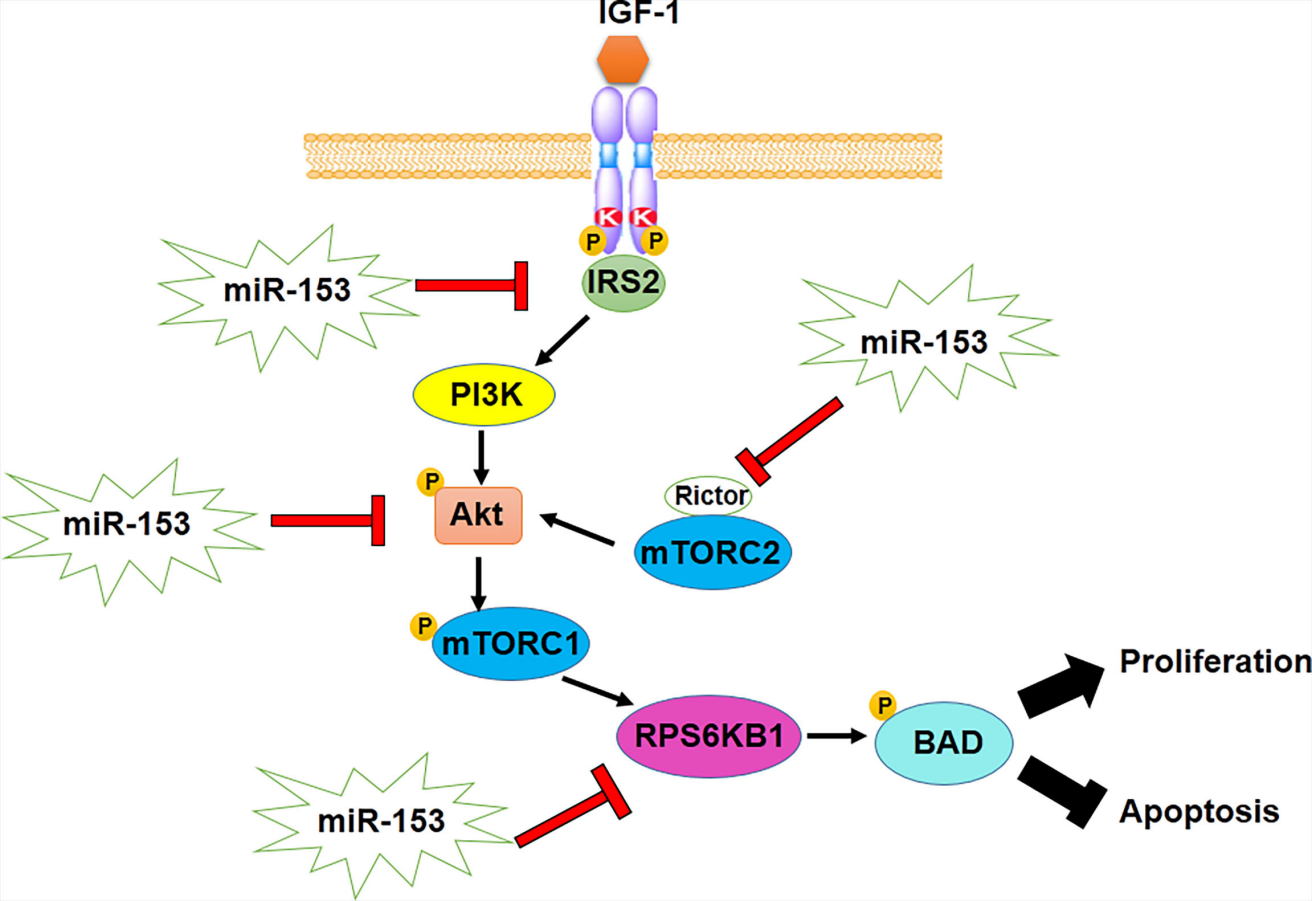
anti-proliferative activity of miR-153 by modulating IRS2/PI3K/AKT/mTOR axis.

Proliferation of cancer cells is mediated by different genes and mechanistic pathways. miR-153, as a tumor suppressor gene, is able to regulate genes related to proliferation (**Table 1**). Down-regulation of miR-153 has been verified in proliferation of various types of cancer, whereas, up-regulation of miR-153 inhibits proliferation through targeting molecules involved in survival and proliferation and modulating some suggested mechanisms (75). FYN, a tyrosine-protein kinase, implicated in cell proliferation and metastasis, is downregulated by miR-153. Downregulation of miR-153 results in overexpression of FYN, promoting proliferation and metastasis of esophageal squamous cell carcinoma (62). Also, the expression level of AKT increases in some types of cancer such as lung cancer, ovarian cancer and glioblastoma. The studies have revealed that an increase in AKT expression can be contributed to downregulation of miR-153. However, overexpression of miR-153 induces cell cycle arrest in G0/G1 and G2/M phases as well as inhibiting proliferation by regulating the expression of AKT, CDK1 through binding to and degrading their mRNAs (3, 23, 24, 26). Moreover, miR-153 suppresses the AKT signaling pathway in glioblastoma through reduction in expression of insulin receptor substrate-2 (Irs-2) which acts as a molecular adaptor, mediating influences of insulin and insulin-like growth factor 1 (IGF-1) on cell proliferation (**Figure 1**) (27). miR-153 inhibits the proliferation of glioma cells by targeting AKT which is also well known as a mTOR complex 2 (mTORC2) indicator (25). In addition, high expression levels of an essential subunit of mTORC2, Rictor, along with overexpression of mTORC2 have been reported in some cancer cells such as glioma cells, whereas miR-153 as an anti-Rictor is downregulated in these cells. Therefore, modulating the mTOR pathway contributes to anti-tumorigenicity activity of miR-153. Furthermore, ribosomal protein S6 kinase B1 (RPS6KB1) is a serine/threonine kinase in downstream of mTOR pathway, implicated in cell viability and inhibition of apoptosis through phosphorylating BAD protein in thyroid carcinoma. miR-153 is involved in inhibiting proliferation and development of thyroid carcinoma by targeting RPS6KB1 in the mTOR-dependent pathway (28). Furthermore, miR-153 regulates the expression level of an E3 ubiquitin ligase, Zinc and ring finger 2 (ZNRF2), which is implicated in the activity of PI3K/Akt/mTOR pathway in papillary thyroid cancer. It recommends the miR-153/ZNRF2 axis, as one of the most important mechanistic pathways in suppressing the PI3K/Akt/mTOR (29).

**Table 1:** Tumor suppressor role of miR-153 in different cancer cells.

Cancer feature	Type of miR-153 transcript	miR-153 Expression	Target gene/protein	Type of cancer	Reference
Proliferation	miR-153-5p	Decreased	AKT	Lung cancer	(3, 23)
				Ovarian cancer	(24)
				Glioma	(25)
	miR-153-5p		CDK1	Breast cancer	(26)
	miR-153-5p		Irs2	Glioblastoma	(27)
	miR‐153‐3p		RPS6KB1	Thyroid carcinoma	(28)
	miR‐153‐3p		ZNRF2	Papillary thyroid cancer	(29)
	miR-153-5p		KLF5	Gastric cancer	(12)
				Breast cancer	(30)
	miR‐153‐3p		E2F3	Thyroid cancer	(31)
	miR-153-5p		TGFβ	Osteosarcoma	(32)
	miR‐153‐3p		ZBTB2	Gastric cancer	(33)
Apoptosis	miR-153-5p	Decreased	BCL-2	Glioblastoma,	(34)
	miR‐153‐3p		BCL-2	CML	(35)
	miR‐153‐3p		MCL-1	Ovarian cancer	(36)
	miR‐153‐5p		MCL-1	Glioblastoma	(34)
	miR‐153‐5p		TGFβ	Nasopharyngeal cancer	(37)
	miR‐153‐5p		HECTD3	Breast cancer	(38)
	miR‐153‐5p		XIAP	AML	(39)
Autophagy	miR‐153‐3p	Decreased	BCL-2	CML	(35)
	miR‐153‐5p		ATG5	Osteosarcoma	(40)
Invasion, Metastasis & EMT	miR‐153‐5p	Decreased	TGFβR2	Breast cancer	(41)
	miR‐153‐5p		Jagged 1	Non-small cell lung cancer	(42)
	miR‐153‐5p		ZEB2	Breast cancer	(43)
				Oral cancer	(44)
				Human epithelial cancer	(44)
				Ovarian cancer	(45)
	miR‐153‐3p		SNAI1	Melanoma	(46)
	miR‐153‐5p		SNAI1	Esophageal squamous cell carcinoma	(47)
				Oral cancer	(44)
				Osteosarcoma	(48)
				Human epithelial cancer	(44)
				Pancreatic adenocarcinoma	(49)
				Laryngeal squamous cell carcinoma	(50)
	miR‐153‐5p		ARHGAP18	Hepatocellular carcinoma	(51)
	miR‐153‐3p		Rabl3	Hepatocellular carcinoma	(52)
	miR‐153‐3p		ROCK1	Breast cancer	(53)
	miR‐153‐3p		Snail	Melanoma	(54)
				Oral carcinoma	(55)
	miR‐153‐5p		Snail	Hepatocellular carcinoma	(56)
	miR‐153‐3p		Snail	Liver cancer	(57)
	miR‐153‐5p		S100A14	Non-small cell lung cancer	(58)
	miR‐153‐3p		MCL-1	Oral cancer	(59)
				Liver cancer	(57)
	miR‐153‐3p		ZBTB2	Gastric cancer	(33)
	miR‐153‐5p		RUNX2	Breast cancer	(60)
	miR‐153‐5p		KIF20A	Cervical cancer	(61)
	miR‐153‐3p		FYN	Esophageal carcinoma	(62)
	miR‐153‐5p		ADAM19	Non-small cell lung cancer	(63)
	miR‐153‐5p		MTDH	Breast cancer	(64)
	miR‐153‐5p		AKT	Glioma	(65)
Angiogenesis	miR‐153‐5p	Decreased	IDO1	Bladder cancer	(66)
	miR‐153‐5p		ANG1	Breast cancer	(67)
	miR‐153‐5p		VEGFA, cdc42	Glioma	(68)
	miR‐153‐5p		IGF-1R	VSMCs	(69)
Radiotherapy resistance	miR‐153‐5p	Decreased	Snail	Pancreatic cancer	(70)
	miR‐153‐5p		JAG1	Pancreatic cancer	(71)
Chemotherapy resistance	miR‐153‐5p	Decreased	ABCE1	Lung cancer	(72)
	miR‐153‐3p		CITED2	Gastric cancer	(73)
	miR‐153‐3p		NRF2	Esophageal squamous cell carcinoma	(74)
	miR‐153‐5p		NRF2	Glioma stem cells	(15)
	miR‐153‐5p		XIAP	AML	(39)
	miR‐153‐3p		BCL2	CML	(35)

Also, anti-proliferative activity of miR-153 is mediated by targeting Kruppel-like factor 5 (KLF5) in breast and gastric cancers. KLF5 is a transcription activator of genes involved in proliferation that is activated by the MEK/ERK1/2 signaling pathway (12, 30). Moreover, upregulation of other transcription factors such as Zinc finger and BTB domain-containing protein 2 (ZBTB2) and RUNX2 due to downregulation of miR-153 may also affect tumorigenicity of gastric cancer. ZBTB2 is a novel partner of the nucleosome remodeling and deacetylase (NuRD) complex which is positively correlated with cancer cell proliferation and metastasis (33). Also, overexpression of RUNX2, a transcription factor involved in regulation of genes related to EMT, metastasis and development of cancer cells, is associated with downregulation of miR-153 in breast cancer cells (60). It suggests that ZBTB2 and RUNX2 may be considered as targets of miR-153, respectively. On the other hand, it has also been confirmed that miR-153 decreases proliferation and promotes cell cycle arrest by targeting E2F3, resulting in downregulation of E2F family transcription factors and Ki67 in thyroid cancer (31).

The other target gene of miR-153, TGFβ, is up-regulated in cancer cells such as osteosarcoma, resulting in overexpression of p-SMAD2, p-SMAD3, EGFR, IGF binding protein-3 (IGFBP-3) which are the crucial proteins in downstream of TGFβ signaling pathway. Therefore, upregulation of miR-153 suppresses proliferation of cancer cells through modulating TGFβ, negatively, which leads to low expression of its downstream proteins (32). It shows that miR-153 acts as an anti-proliferative factor which is downregulated in various types of cancer. Totally, it may be introduced as a novel biomarker to detect cancer as well as usage as a novel strategy for cancer therapy.

## Role of miR-153 in apoptosis

Recently, the studies have shown the pro-apoptotic role of miR-153 in cancer cells (**Table 1**) (14, 76). It has been reported that the low rate of apoptosis in some cancer cells, including glioblastoma, ovarian cancer, CML and AML, may be contributed to downregulation of miR-153 which targets three antiapoptotic genes, *Bcl-2*, *Mcl-1* and *XIAP* through binding to 3´UTR of their mRNAs (34, 36, 39). Recently, it has been shown that *Mcl-1* is also well known as a miR-153 target gene in inhibiting metastasis in some types of cancer, including oral and liver cancer (57, 59). Thus, besides the anti-apoptotic role, MCL-1 plays an important activity in migration, metastasis and development of a variety of cancer cells. In addition, it has been confirmed that miR-153 has a critical role in cell viability reduction and apoptosis induction through activating caspase 3, 9 and rising Bax/Bcl2 ratio, which are mediated by targeting TGFβ/Samd2 axis in nasopharyngeal cancer cells (37). On the other hand, miR-153 is considered as a negative regulator of homologous to the E6-associated protein carboxyl terminus domain containing 3 (HECTD3), an E3 ubiquitin ligase. HECTD3 promotes ubiquitination of some types of caspases, including caspase 8 and 9, resulting in apoptosis inhibition and cell survival promotion in cancer cells such as breast cancer. Thus, modulating HECTD3 is implicated in miR-153-induced apoptosis in these cancer cells (38). Besides apoptotic activity, miR-153 regulates autophagy in CML and osteosarcoma. miR-153 inhibits autophagy by downregulating Bcl2 as well as targeting ATG5 (35, 40). Therefore, it can be recommended as a therapy strategy through stimulating apoptosis and suppressing autophagy.

## Role of miR-153 in invasion and metastasis of cancer cells

miR-153 regulates invasion, metastasis and progression of different types of cancer through some suggested mechanisms. Numerous studies have verified that downregulation of miR-153 increases migration and metastasis of some types of cancer cells, whereas upregulation of miR-153 suppresses invasion and metastatic phenotypes of cancer cells (**Table 1**) (41, 43, 46, 54, 74). In addition, the role of miR-153 has been shown in inhibiting stemness phenotypes of cancer stem cells (CSCs) such as self-renewal, proliferation and tumorigenicity of lung cancer cells (42). Jagged1/Notch, one of the signaling pathways involved in CSCs, is regulated by miR-153 through targeting Jagged 1, Notch1 ligand in non-small cell lung cancer (42). Thus, Jagged1/Notch can be considered as targets for lung cancer treatment modulated by miR-153.

**Figure 2:**
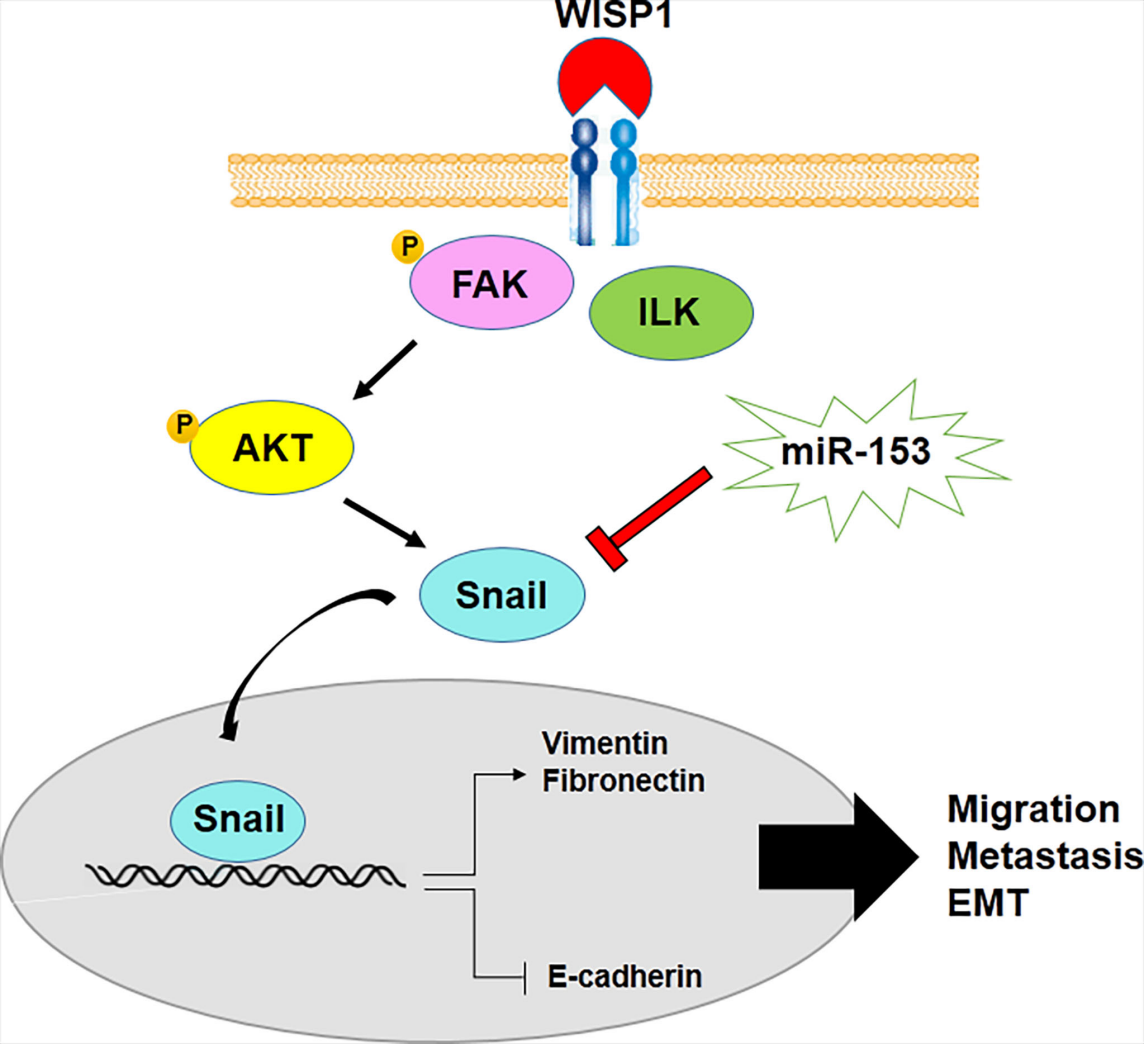
miR-153 regulates metastasis and EMT of cancer cells through decrease in E-cadherin and increase in expression levels of mesenchymal markers.

On the other hand, epithelial mesenchymal transition (EMT), which is a tumor progression process in malignant cancer cells and CSCs, is regulated by miR-153. Downregulation of miR-153 leads to the development of EMT through decrease in E-cadherin, the epithelial marker, as well as an increase in expression levels of mesenchymal markers, including vimentin, Snail and fibronectin. Overexpression of mesenchymal markers is mediated by upregulation of *SNAI1*, one of the other miR-153 target genes in various types of cancer cells such as melanoma, esophageal squamous cell carcinoma, oral cancer, osteosarcoma, human epithelial cancer, pancreatic adenocarcinoma and laryngeal squamous cell carcinoma (48, 50, 54, 56, 57, 77). miR-153 suppresses SNAI1-induced metastasis through negative regulation of SNAI1 in some types of cancer (44, 46, 47, 49). Also, WNT1-inducible signaling pathway protein-1 (WISP1) inhibits miR-153-induced downregulation of Snail mRNA translation and promotes migration, metastasis and EMT of oral squamous cell carcinoma cells (**Figure 2**) (55). Therefore, it demonstrates WISP1/miR-153/Snail axis acts as an important pathway in EMT and metastasis of malignancies.

One of the other suggested molecular mechanisms involved in anti-metatstatic activity of miR-153 is mediated by targeting TGFβ receptor 2 (TGFβR2) that leads to inhibit invasion, migration and EMT in breast cancer (41). In addition, metadherin (MTDH), a new target of miR-153, is an oncogene inducing EMT through activating PI3K/Akt, Wnt/β-catenin, MAPK and NFκB signaling pathways. Therefore, these signaling pathways may be recognized as indirect targets of miR-153, which are over activated by downregulation of miR-153 in cancer cells (64, 78). It recommends the miR-153/MTDH axis as a prognostic biomarker and a novel targeted therapy for treatment of breast cancer. Also, it has been shown that expression of miR-153 is closely correlated with decreased expression of p-Akt in the PI3K signaling pathway regulating invasion and metastasis of glioma cells (65).

Furthermore, anti-tumorigenicity activity of miR-153 contributes to aberrant expression of a transcriptional repressor of E-cadherin coding gene, ZEB2, which plays a critical role in metastasis and EMT (43, 44). In addition, the expression of RhoGTPase activating protein 18 (ARHGAP18), Rab-like protein 3 (Rabl3) and Rho associated coiled-coil containing protein kinase 1 (ROCK1), which play a vital role in migration and metastasis of cancer cells, are negatively regulated by miR-153 in hepatocellular carcinoma and breast cancer (51–53). Kinesin-like protein (KIF20A) is another target of miR-153 which is responsible for intracellular organelle transport and cell migration. miR-153 promotes downreulation of KIF20A and inhibits migration and invasion of cervical cancer cells (61). Also, S100A14, a calcium dependent regulatory protein with a phosphorylation site by protein kinase C (PKC), is one of the other miR-153 targets which is closely related to proliferation and metastasis of non-small cell lung cancer cells (58). Furthermore, degradation of extracellular matrix proteins by A Disintegrin and Metalloproteinase (ADAM) proteolytic enzymes is a key step to migration and metastasis of cancer cells. Downregulation of miR-153 is correlated with overexpression of *ADMA19*, proposing *ADMA19* as another miR-153 target gene in non-small cell lung cancer (63). Totally, miR-153 suppresses migration, invasion, EMT and metastasis of cancer cells through some suggested pathways, resulting in introducing miR-153 as a prognosis marker and therapy target for malignancies.

## Role of miR-153 in angiogenesis of cancer cells

**Figure 3:**
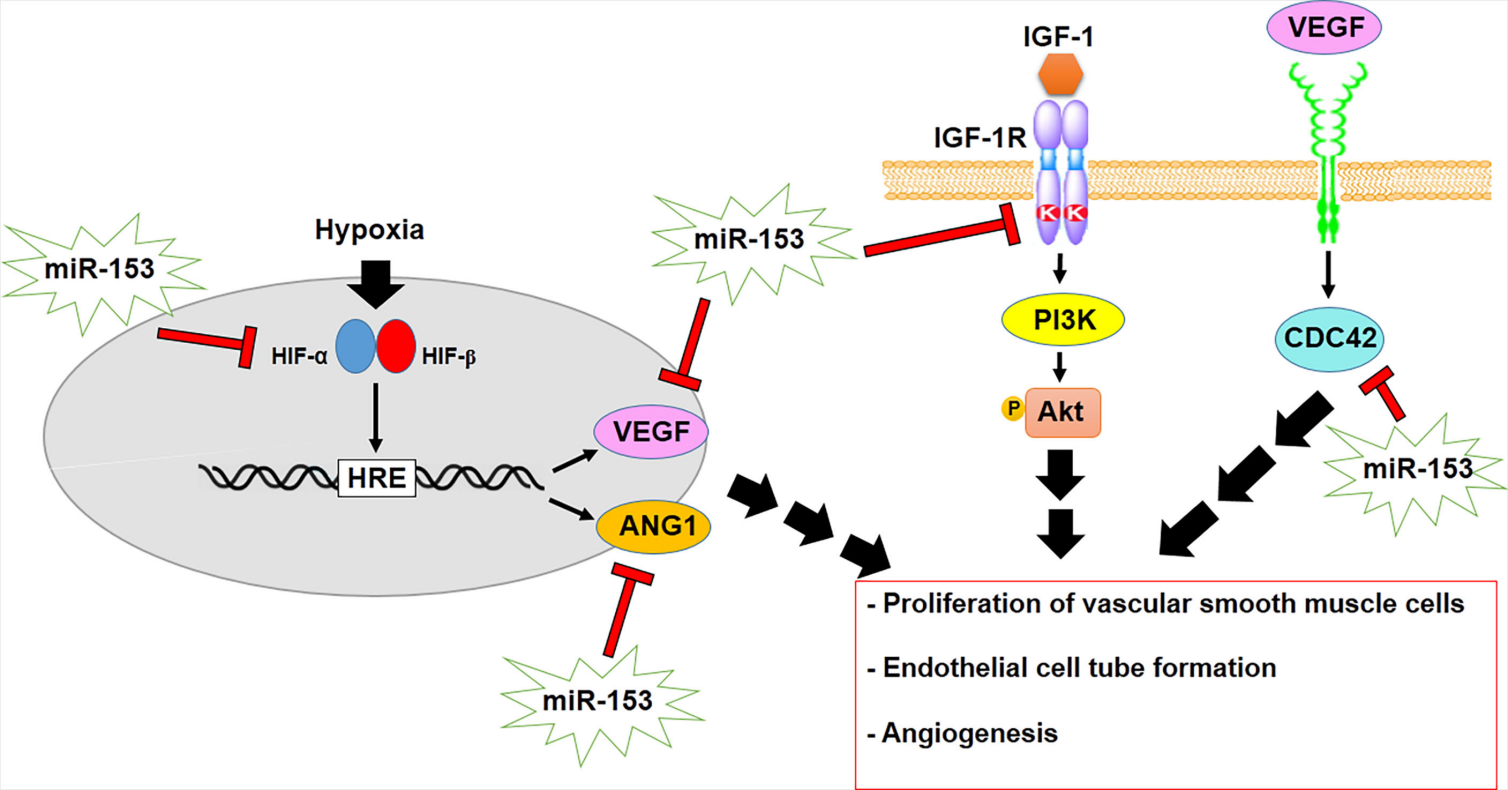
miR-153 regulates angiogenesis and proliferation of VSMCs by modulating HIF1α/VEGF/CDC42 axis as well as inhibiting PI3K/Akt signaling.

Angiogenesis is a vital process in the development of tumors that is modulated by hypoxia and thus, hypoxia-induced HIF1 mediates angiogenesis in cancer cells. HIF1α promotes the expression of some angiogenic factors, including vascular endothelial growth factor (VEGF) and angiopoietin 1 (ANG1) by binding to HIF1 response element (HRE) (79). Recently, it has been verified that miR-153, as a tumor suppressor gene, regulates angiogenesis by modulating VEGF and ANG1 in glioma and breast cancer, respectively (**Table 1**). miR-153 suppresses angiogenesis through targeting HIF1α as well as negative regulation of ANG1 and inhibition of endothelial cell tube formation and migration in breast cancer (**Figure 3**) (67, 80, 81). On the other hand, miR-153 declines the expression of angiogenesis promoting factors such as VEGFA and cdc42 by binding to 3´UTR of their mRNAs in glioma (68). Therefore, it indicates that miR-153 regulates HIF1α/VEGF axis in angiogenesis, proposing usage as an anti-angiogenesis therapy in cancer. In addition, the insulin-like growth factor1 receptor (IGF-1R) mediates proliferation of vascular smooth muscle cells (VSMCs) through promoting the PI3K/Akt signaling pathway in hyperplasia. miR-153 suppresses proliferation of VSMCs by targeting and downregulating IGF-1R as well as inhibiting PI3K/Akt signaling pathway (69).

Furthermore, high metabolism in cancer cells is related to malignancy of cancer cells. Increased metabolism of tryptophan has been observed in angiogenesis. Metabolism of tryptophan is mediated by indoleamine 2,3-dioxygenase 1 (IDO1), which is downregulated by miR-153 in bladder cancer. Therefore, miR-153 can reduce tryptophan metabolism and also modulate angiogenesis through suppressing the IL6/STAT3/VEGF signaling pathway (66). The studies have confirmed that miR-153 may be used as a prognostic marker for angiogenesis and applied as a target for treatment of a variety of malignancies.

## Role of miR-153 in radio and chemotherapy resistance of cancer cells

**Figure 4:**
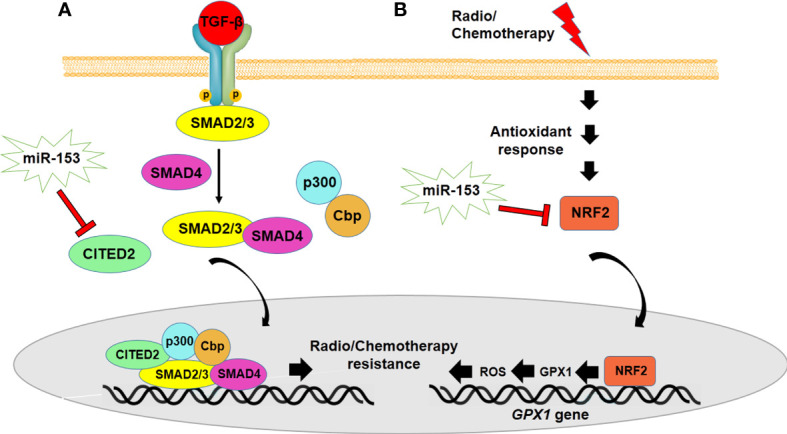
miR-153 regulates radio/chemotherapy resistance by modulating expression of CITED2 **(A)** and NRF2 **(B)**. **(A)** miR-153 targets CITED2 which is implicated in drug resistance of cancer cells through regulating TGF-β signaling. **(B)** NRF2 overexpression controls the expression of glutathione peroxidase 1 (GPX1) and reduces ROS which leads to enhancement of chemotherapy resistance.

Some potential mechanisms are implicated in radio/chemotherapy resistance ability of cancer cells and CSCs mediating recurrence in some types of cancer (82). Recently, numerous studies have reported the role of miRNAs, in particular, miR-153, in regulating radio and drug resistance ability of cancer cells (**Table 1**) (70, 71, 83, 84). The expression of miR-153 is dysregulated in radiotherapy resistant cancer cells. However, high expression level of miR-153 increases sensitivity of cancer cells to radiotherapy, which may be mediated by targeting Snail and Jagged canonical notch ligand1 (JAG1) in pancreatic cancer (70, 71). Moreover, downregulation of miR-153 has been reported in drug resistant cancer cells, whereas miR-153 overexpression enhances the sensitivity of cancer cells to chemotherapy (26, 35, 76). One of the suggested mechanisms of drug resistance of cancer cells is upregulation of ABCE1 due to downregulation of miR-153 in lung cancer cells (72). Therefore, *ABCE1* is suggested as miR-153 target gene modulating drug resistance of cancer cells. In addition, miR-153 regulates drug resistance by modulating expression of Cbp/p300-interacting transactivator 2 (CITED2), which is implicated in drug resistance of gastric cancer cells (**Figure 4**). CITED2 regulates the TGF-β signaling pathway through its association with the SMAD/p300/CBP-mediated transcriptional coactivator complex (73). Also, miR-153 is able to regulate the nuclear factor erythroid 2-related factor 2 (NRF2), a transcription factor upregulated in antioxidant responses of radio/chemotherapy resistant cancer cells. NRF2 overexpression controls the expression of glutathione peroxidase 1 (GPX1) and reduces ROS, which leads to enhancement of chemotherapy resistance (15). However, aberrant expression of NRF2 as a result of upregulation of miR-153, increases sensibility of cancer cells and CSCs to radio/chemotherapy in some types of cancer cells including esophageal squamous cell carcinoma and glioma stem cells (15, 74). In addition, miR-153 decreases drug resistance of AML through targeting XIAP, which is responsible for protecting cells from apoptosis by blocking the function of caspases (39). Recently, miR-153 has been recommended as an adjuvant in treatment of various types of leukemias. Acute promyelocytic leukemia (APL) and Chronic myeloid leukemia (CML) show drug resistance to As_2_O_3_, which is well-known as an effective drug in the treatment of APL, CML and other leukemias (85). The expression of miR-153 reduces in As_2_O_3_-resistant CML, whereas overexpression of miR-153 induces greater apoptosis in combination with As_2_O_3_ in CML (76). Also, downregulation of miR-153 has been observed in drug resistance ability of CML to imatinib. However, miR-153 upregulation increases sensitivity of CML to chemotherapy through downregulating Bcl2, resulting in autophagy reduction in drug resistant CML (35). It indicates that miR-153 overexpression in combination with chemotherapy may be used as a novel strategy to combat with leukemias. Totally, the studies suggest that miR-153 can be used as a molecular target in order to reduce the drug resistance of cancer cells.

## Oncogenic roles of miR-153 in cancer cells

Besides tumor suppressor activity of miR-153 in various types of cancer, the oncogenic role of miR-153 has been confirmed in some studies (16, 17, 86). There is a positive correlation between the overexpression of miR-153 and aggressive phenotypes of cancer cells including, proliferation, lymph node metastasis, EMT and drug resistance that can be mediated by targeting some suggested target genes (**Table 2**). Proliferative and metastatic activity of miR-153 is mediated by activating the PI3K/Akt signaling pathway. One of the suggested mechanisms involved in the oncogenic role of miR-153 is downregulating PTEN, which is responsible for suppressing the PI3K/Akt signaling pathway in prostate cancer cells (17). miR-153 promotes proliferation of prostate cancer cells through enhancing G1/S phase transition, cyclin D expression as well as inhibiting CDK inhibitor (p21) that are mediated by targeting PTEN. Aberrant expression of PTEN results in activation of PI3K/Akt signaling and downregulation of Forkhead box class O 1 (FOXO1) in these types of cancer cells (86, 87). Also, the expression of Argonaute RISC catalytic component 1 (AGO1), a miRNA processing protein in RISC complex, is downregulated in renal cell carcinoma due to upregulation of miR-153. The miR-153/AGO1 axis induces progression of renal cell carcinoma through activating PI3K/Akt signaling pathway. It recommends both AGO1 and miR-153 as potential prognostic markers for detecting this type of cancer (16). One of the other signaling pathways involved in tumorigenicity activity of miR-153 is Wnt/β catenin signaling pathway which is activated by miR-153 targeting WWOX, a tumor suppressor and inhibitor of Wnt/β catenin in hepatocellular carcinoma (88). Moreover, it has been reported that miR-153 is upregulated in triple negative breast cancer and may exhibit an oncogenic role in breast cancer development and progression. miR-153 inhibitor suppresses proliferation and induces apoptosis through enhancing caspase 3/7 activity in triple negative breast cancer (89). The oncogenic role of miR-153 is associated with an increase in anti-apoptotic proteins such as surviving and BCL2, as well as a decrease in p21. miR-153 promotes proliferation and metastasis of cancer cells such as hepatocellular carcinoma through suppressing the efficiency of chemical and targeted drugs including Etoposide, Paclitaxel and Sorafenib (86). miR-153-induced dug resistance of cancer cells is promoted by production of matrix metalloproteinase (MMP9) as well as inhibiting transcription factor Forkhead box class O 3a (FOXO3a), a tumor suppressor protein in colorectal cancer (18). Totally, miR-153 can be considered as an oncogene and potential prognostic marker for detecting some types of cancer that may be suggested as a novel therapy target.

**Table 2:** Oncogenic role of miR-153 in different cancer cells.

Cancer feature	Type of miR-153 transcript	miR-153 expression	Target gene/protein	Type of cancer	Reference
Proliferation	miR‐153‐5p	Increased	AGO1	Renal cell carcinoma	(16)
	miR‐153‐5p		PTEN	Prostate cancer	(17, 87)
	miR‐153‐5p		WWOX	Hepatocellular carcinoma	(86) (88),
Metastasis	miR‐153‐5p	Increased	PTEN	Prostate cancer	(17)
	miR‐153‐5p		AGO1	Renal cell carcinoma	(16)
Drug resistance	miR‐153‐5p	Increased	FOXO3a	Colorectal	(18)

## Controversial role of miR-153 as both tumor suppressor and oncogenic miRNA

**Figure 5:**
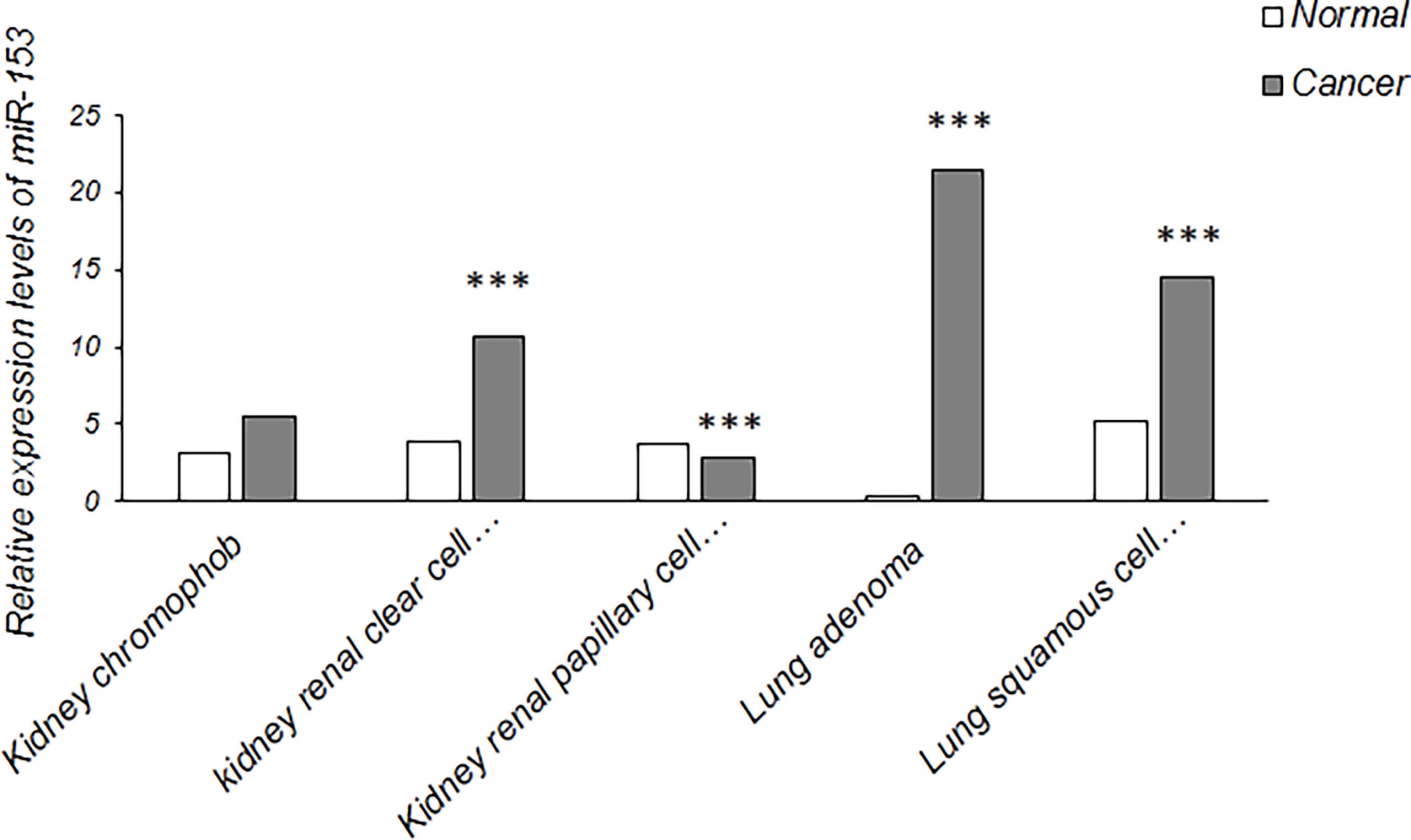
Relative expression levels of miR-153 in some cancers with diverse histological subtypes (The data were obtained from ENCORI). Kidney chromophob (N = 65) against normal samples (N = 24), kidney renal clear cell carcinoma (N = 517) against normal samples (N = 71), kidney renal papillary cell carcinoma (N = 289) against normal samples (N = 32), lung adenocarcinoma (N = 512) against normal samples (N = 20) and lung squamous cell carcinoma (N = 475) against normal samples (N = 38). N indicates the number of tissue samples. ***P < 0.001.

It has been confirmed that miR-153 acts as both tumor suppressor gene and oncogene which may be dependent on type of cancer cells with different gene expression profiles (17). It proposes a tumor suppressive role of miR-153 which is downregulated in most types of tumors including non-small cell lung cancer, liver cancer, glioblastoma, thyroid carcinoma, gastric cancer, osteosarcoma, melanoma, oral cancer, pancreatic adenocarcinoma, bladder cancer, cervical cancer and leukemias, and an oncogenic activity of miR-153 which is upregulated in prostate, colorectal and renal cell carcinoma. In renal cell carcinoma, the signaling pathway involved in oncogenic effect of miR-153 is mainly PI3K/AKT signaling pathway which has a critical role in cell cycle progression and proliferation by upregulation of cyclin D1 as well as EMT-mediated metastasis by downregulation of E-cadherin and upregulation of Snai1. Also, PI3K/AKT signaling pathway is suppressed by AGO1, a tumor suppressor gene and one of the miR-153 targets which is implicated in gene silencing and translational inhibition of mRNAs (16). miR-153 has also an oncogenic role in prostate cancer. One of the signaling pathways promoting prostate cancer is PI3K/AKT signaling pathway which is regulated by PTEN, a PI3K/AKT signaling inhibitor. PTEN is frequently downregulated in prostate tumors that may be mediated by upregulation of miR-153. miR-153 targets PTEN to activate PI3K/AKT signaling pathway, resulting in cell cycle progression and proliferation through upregulation of cyclin D1 and downregulation of p21, a CDK inhibitor (87). Moreover, in colorectal cancer, higher expression of miR-153 has been detected in primary cancer cells compared with normal colonic epithelial cells and in advanced stage of tumor compared with early stages. It has been revealed that miR-153 supports colorectal cancer *via* pleiotropic effects that enhance progression and chemotherapeutic resistance (18). Compared to early stages of colorectal cancer, overexpression of MMP9 is strongly associated with more advanced stages. It has been confirmed that miR-153 upregulates MMP9 through targeting some predicted transcription factors that may suggest mechanism for this process (18). In addition, it is assumed that miR-153 has a controversial role as both tumor suppressive and oncogenic miRNAs in hepatocellular carcinoma and breast cancer in a manner dependent on heterogeneity and tumor stage. In early clinical stages, miR-153, as an oncogenic miRNA, promotes proliferation and tumor growth of hepatocellular carcinoma through modulating β-catenin signaling pathway (88). On the other hand, it acts as tumor suppressor miRNA in metastatic cancer and in an advanced clinical stage of hepatocellular carcinoma by targeting ARHGAP18, Rabl3 and snail which are up-regulated and implicated in migration and metastasis of this type of cancer cells (51, 52, 56). Moreover, abnormal downregulation of miR-153 can also be associated with advanced clinical stage of breast cancer as detected in human ovarian tumor (89, 90). Also, the expression of a specific miRNA may be different due to diverse histological subtypes in a certain cancer (91). For example, data from The Encyclopedia of RNA Interactomes (ENCORI) showed that the expression levels of miR-153 in kidney chromophob is higher than kidney renal papillary cell carcinoma and is lower than kidney renal clear cell carcinoma. The expression levels of miR-153 is also higher in lung adenocarcinoma compared with lung squamous cell carcinoma (**Figure 5**). Further investigation is recommended to detect miRNA expression in different histological subtypes of other cancers.

**Figure 6:**
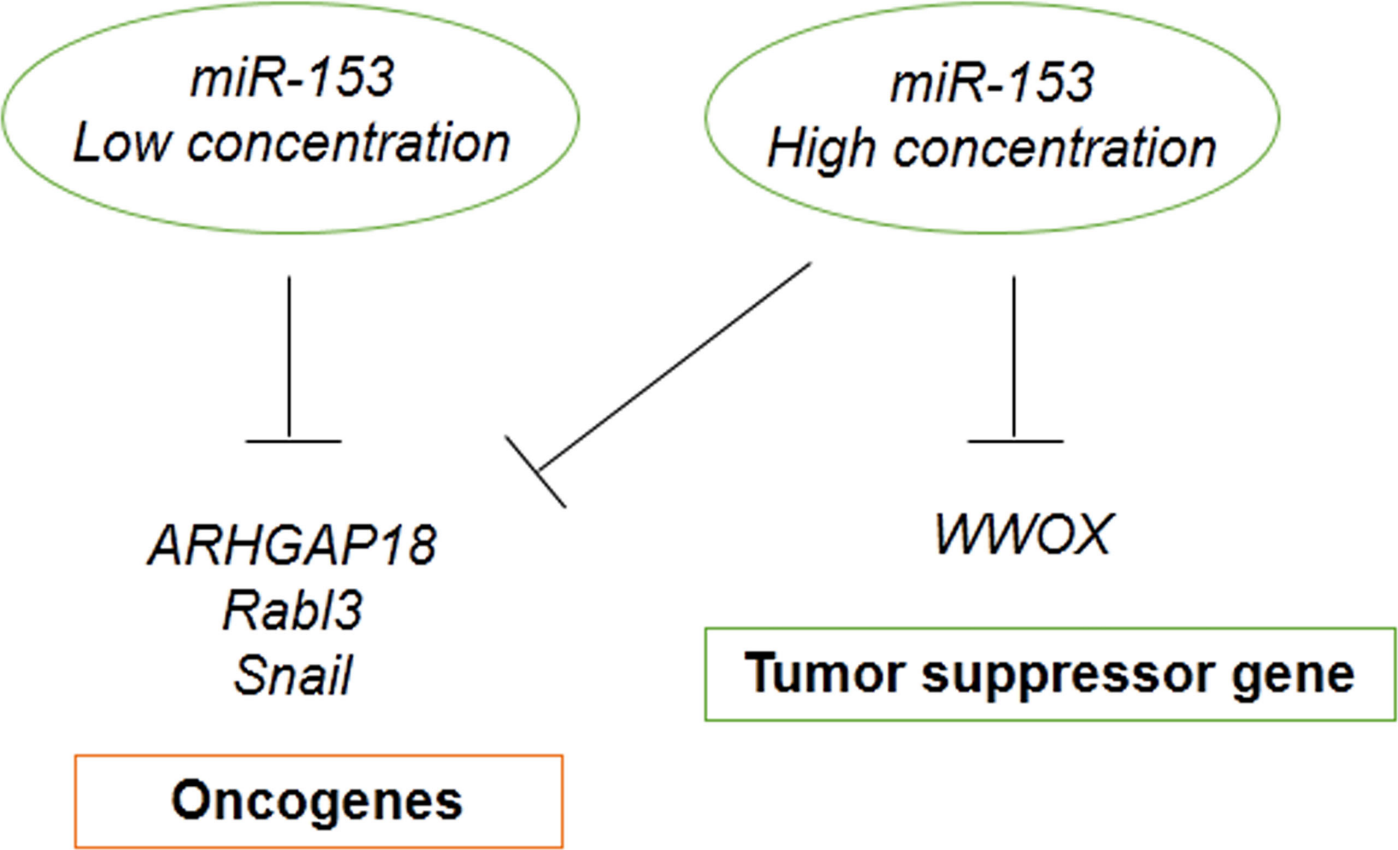
Dual role of miR-153 may be contributed to miRNA concentration and targets abundance.

In addition, miRNA concentration affect gene silencing. Some target genes require lower miRNA concentration and some others need higher miRNA abundance for silencing that depends on miRNA-mRNA complementary base pairing (92). It is suggested that the concentration of miR-153 is one of the other reasons causing the different effects of miR-153. Because oncogenes are more abundant targets of miR-153, they require low concentrations of miRNA to be silenced. On the other hand, tumor suppressor genes are less abundant targets of miR-153, they require high concentrations of miRNA to be silenced. Therefore, miR-153 has the tumor suppressive and oncogenic roles in the low and high concentrations, respectively (**Figure 6**).

To date, many miR-153 target genes have been confirmed, and there are numerous other unverified genes that can be regulated by miR-153 and affect several signaling pathways, simultaneously. Also, a specific target can be regulated by various miRNAs. Therefore, it influences the ultimate function of miRNA, just as the expression level of the target genes also affects the function of miRNA. In a study, tumor suppressive and oncogenic effects of miR-204 have been confirmed through targeting XRN1 in prostatic adenocarcinoma cells and neuroendocrine-like prostate cancer cells, respectively (93). In another report, inhibition of all miRNAs targeting FOXO1 in endometrial cancer cells with low levels of FOXO1 expression (Ishikawa cells) induced cell cycle arrest, while no significant changes were observed in endometrial cancer cells with high levels of FOXO1 (HEC-1B cells) (94). Therefore, contradictory role of miRNAs in cancer may be due to the variable expression levels of the target genes in cancer cells. It has been shown that miR-153 induces apoptosis through targeting HECTD3 in BT-549 breast cancer cells, whereas in another report miR-153 inhibits apoptosis through indirect targeting of caspase 3/7 activity in triple negative breast cancer (38, 89). Ultimately, the function of miRNA is determined by the network of these target genes with different expression and functions (92). The abundance of these targets with opposite function may be contributed to the dual role of miR-153 in cancers.

Furthermore, the dual role of miR-153 in cancer may also be contributed to single nucleotide polymorphisms (SNPs) in miRNAs or miRNA-binding sites (92). SNPs can affect binding affinity of miRNAs to their target genes. For example, it has been reported that there is a significant association between SNP-rs66461782 in miR-186 and breast cancer. On the other hand, rs1062577 on its target gene, ESR1, decreased the binding affinity of miR-186 to ESR1 mRNA due to alteration in the number of hydrogen bonds (92). Several SNPs in pre-miR-153-5p, pre-miR-153-3p and three SNPs on the seed site of miR-153-3p (rs747400621, rs1478975893 and rs1034944138) have been recognized (http://bioinfo.life.hust.edu.cn/miRNASNP/#!/) that may affect the biogenesis and function of miR-153 on its target genes. There is still no evidence that has revealed the association between these SNPs with various cancers. Furthermore, there are numerous SNPs on the 3´UTR of target genes of miR-153 that may create or disturb negative regulatory effects of miR-153 on target mRNAs. For example, there is a significant association between 3′UTR WWOX SNP (rs73569323) and risk of hepatocellular carcinoma, as it has shown that T allele decreases risk of hepatocellular carcinoma with an unknown mechanism (95). Also, it has been reported that 3′UTR polymorphisms of amyloid precursor protein (APP) (*APP-118C/A* and *APP-534G/A*) may affect the binding affinity of miR-153 and the regulation of APP expression by this miRNA in Alzheimer’s disease (96). In the other research, it has also been verified that the expression of C/T allele variants (rs6932603) in *CCDC170* may be regulated by miR-153. miR-153 significantly downregulated rs6932603-T expression in bone osteosarcoma epithelial cells (97). Therefore, studies can be extended to identify the association between SNPs in miR-153 and its target genes with different types of cancers as well as detecting effects of these SNPs on the miR-153 binding to its target genes in cancers.

## Non coding RNAs modulating the expression of miR-153 in cancer cells

**Figure 7:**
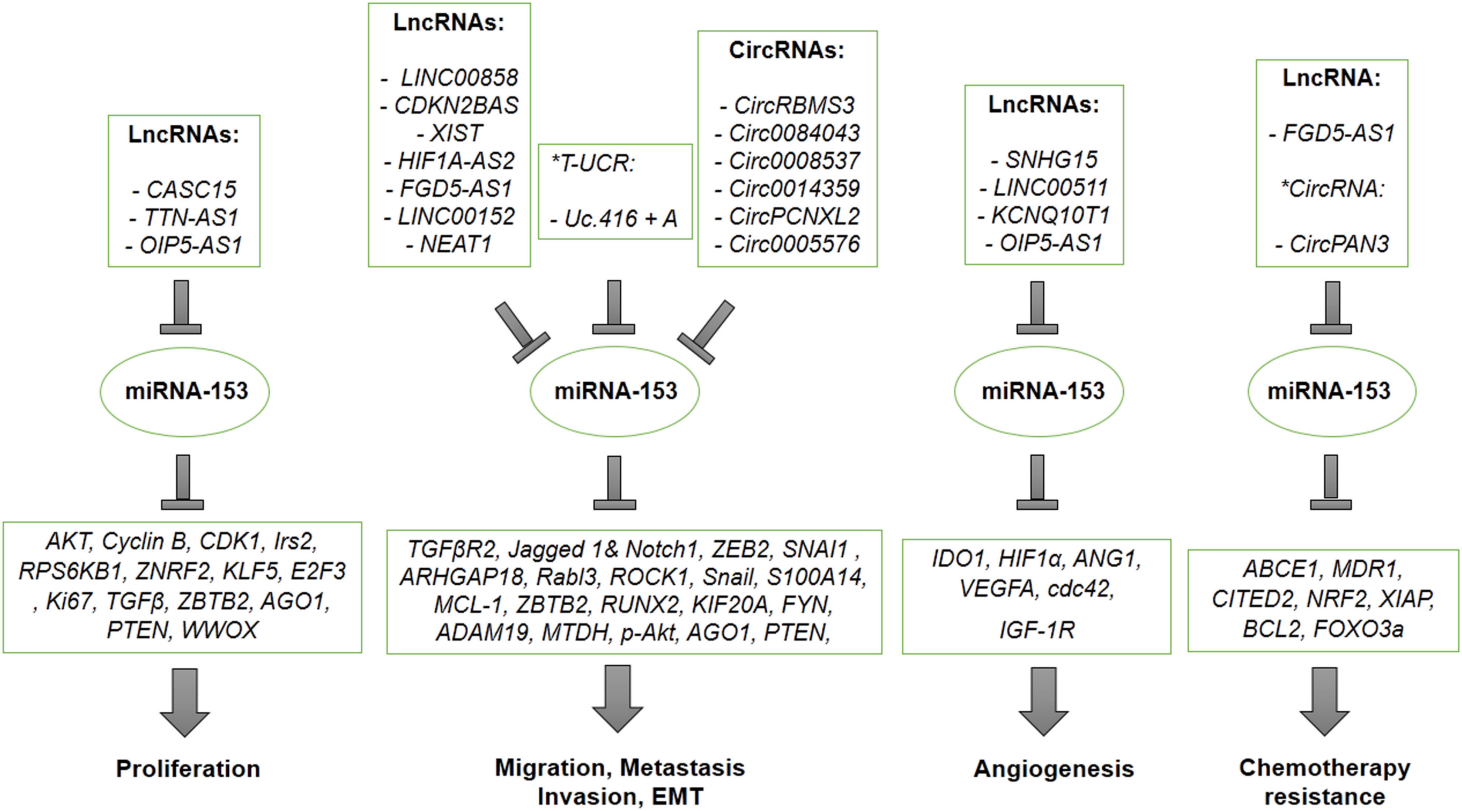
A summary figure for lncRNA-related regulation of miR-153 and an overall schematic representation of miR153 in cancer.

There are various types of non-coding RNAs, including long non-coding RNAs (LncRNAs), transcribed ultra-conserved regions (T-UCRs) and circular RNAs (CircRNAs) regulating miR-153, negatively. Several types of non-coding RNAs are upregulated in various types of cancer cells that results in inhibiting anti-cancerous effects of miR-153. A number of LncRNAs, T-UCRs and CircRNAs play an oncogenic role in proliferation, invasion, metastasis, angiogenesis, and drug resistance ability of cancer cells by targeting and sponging miR-153 through some suggested mechanisms (**Table 3**). **Figure 7** also presents a summary for non-coding RNAs-related regulation of miR-153 and an overall schematic representation of miR153 in cancer. LncRNA CASC15 promotes proliferation of breast cancer cells through sponging miR-153 targeting KLF5 (98). It has been confirmed that LncRNA TTN-AS1 and LncRNA OIP5-AS1 increase proliferation of papillary thyroid cancer and gastric cancer through miR-153/ZNRF2 axis and miR-153/ZBTB2 axis, respectively (29, 33). LncRNA CDKN2BAS and LncRNA LINC00858 induce migration, invasion and metastasis of hepatocellular carcinoma through suppressing miR-153 and upregulating ARHGAP18 and Rabl3 (51, 52). Also, LncRNA HIF1A-AS2 and LncRNA NEAT1 promote migration, invasion and metastasis of non-small cell lung cancer in a manner dependent on the miR-153/S100A14 axis and the miR-153/Wnt signaling axis (58, 78). Moreover, oncogenic activity of LncRNAs including LncRNA-XIST, LncRNA FGD5-AS1, LncRNA LINC00152 is mediated by overexpressing SNAI1, MCL1, FYN in osteosarcoma, oral cancer, esophageal carcinoma, respectively (48, 59, 62).

**Table 3:** Non coding RNAs modulating the expression of miR-153 in different cancer cells.

Non-coding RNA	Target	Tumorigenicity activity	Type of cancer	References
LncRNA CASC15^1^	miR-153-3p/KLF5 axis	Proliferation	Breast cancer	(98)
LncRNA TTN-AS1^2^	miR-153-3p/ZNRF2 axis	Proliferation	Papillary thyroid cancer	(29)
LncRNA OIP5-AS1	miR-153-3p/ZBTB2 axis	Proliferation and metastasis	Gastric cancer	(33)
LncRNA CDKN2BAS	-miR-153-5p/ARHGAP18/axis -Downregulation of KLFB	Migration, Invasion, Metastasis	Hepatocellular carcinoma	(51)
LncRNA LINC00858	miR-153-3p/Rabl3 axis	Migration, Invasion, Metastasis	Hepatocellular carcinoma	(52)
LncRNA-XIST	miR-153-5p/SNAI1 axis	Migration, Invasion, Metastasis	Osteosarcoma	(48)
LncRNA HIF1A-AS2	miR-153-5p/S100A14 axis	Migration, Invasion, Metastasis	Non-small cell lung cancer	(58)
LncRNA FGD5-AS1	miR-153-3p/MCL1 axis	Migration, Invasion, Metastasis	Oral cancer	(59)
LncRNA LINC00152	miR-153-3p/FYN axis	Metastasis	Esophageal carcinoma	(62)
LncRNA NEAT1^3^	miR-153-3p/Wnt signaling axis	Migration, Invasion, Metastasis	Non-small cell lung cancer	(78)
LncRNA SNHG15	miR-153-5p/VEGFA, cdc42 axis	Angiogenesis	Glioma	(68)
LncRNA LINC00511	miR-153-5p/HIF1/ LINC00511 (Positive feedback)	Angiogenesis	Colorectal cancer	(80)
LncRNA KCNQ10T1	miR-153-3p/HIF1	Angiogenesis	Retinoblastoma	(81)
LncRNA OIP5-AS1	miR-153-5p/ATG5	Angiogenesis and autophagy	Osteosarcoma	(40)
Lnc-RNA FGD5-AS1	miR-153-3p/CITED2	Chemotherapy resistance	Gastric cancer	(73)
T-UCR^4^ ncRNA Uc.416 + A	-Upregulation of Vimentin, Snail -Downregulation of E-cadherin (CDH1)	EMT	Renal carcinoma	(77)
CircRBMS3^5^	miR-153-5p/SNAI1 axis	Migration, Invasion, Metastasis	Gastric cancer	(99)
CircRNA-0084043	miR-153-3p/Snail axis	Migration, Invasion, Metastasis	Melanoma	(54)
Hsa-Circ-0008537	miR-153-3p/Snail,Mcl-1 axis	Migration, Invasion, Metastasis	Liver cancer	(57)
Has-Circ-0014359	miR-153-5p/PI3K signaling axis	Migration, Invasion, Metastasis	Glioma	(65)
CircPCNXL2	miR-153-5p/ZEB2 axis	Migration, Invasion, Metastasis	Renal carcinoma	(100)
Circ-0005576	miR-153-5p/KIF20A axis	Migration, Invasion, Metastasis	Cervical cancer	(61)
CircPAN3	miR-153-5p/XIAP axis	Chemotherapy resistance	AML	(39)

^1^Cancer susceptibility candidate 15; ^2^LncRNA TTN antisense RNA 1; ^3^Nuclear enriched abundant transcript; ^4^Transcribed ultraconserved region; ^5^Circular RNA BMS3.

On the other hand, angiogenic activity of LncRNAs has been confirmed by some studies. It has been reported that angiogenic activity of LncRNA SNHG15 is regulated by suppressing miR-153, which targets VEGFA and cdc42 in glioma (68). Furthermore, LncRNAs including LncRNA LINC00511 and LncRNA KCNQ10T1 promote angiogenesis by overexpressing HIF1 in a manner dependent on LncRNAs/miR-153/HIF1 in colorectal cancer and retinoblastoma (80, 81). HIF1 is considered as a potential transcription factor regulating the expression of LncRNA LINC00511. Therefore, it suggests positive feedback for LncRNA LINC00511 as miR-153/HIF1/LINC00511 (80). Also, aberrant upregulation of lncRNA OIP5-AS1 regulates angiogenesis and autophagy of osteosarcoma through down-regulating miR-153, which targets ATG (40).

In addition, resistance to chemotherapy may be mediated by Lnc-RNA FGD5-AS1 suppressing miR-153 and upregulating CITED2, a gene target of miR-153, in gastric cancer (73).

T-UCR ncRNA Uc.416 + A is a non-coding RNA which is well-known as a crucial factor for EMT of cancer cells such as renal carcinoma. Tumorigenic activity of T-UCR ncRNA Uc.416 + A contributed to upregulating vimentin and snail through sponging miR-153 (77).

CircRNAs, the other regulating RNAs, have been recognized as non-coding RNAs which have an important role in tumorigenicity of cancer cells by modulating miRNAs in particular miR-153. CircRNAs such as CircRBMS3, CircRNA-0084043 and Hsa-Circ-0008537 promote cancer cell migration, invasion and metastasis through upregulating SNAI1 and snail as a result of sponging miR-153 in melanoma, gastric cancer and liver cancer (54, 57, 99). One of the other CircRNAs contributing to migration, invasion and metastasis of cancer cells by modulating miR-153 is Has-Circ-0014359, which is upregulated in glioma cells and leads to an increase in the PI3K signaling pathway (65). Also, miR-153 can be targeted by CircPCNXL2, which overexpresses ZEB2 in renal carcinoma in a manner dependent on the CircPCNXL2/miR-153/ZEB2 axis (100). Circ-0005576 is also overexpressed in cervical cancer resulting in upregulation of KIF20A, one of the other target genes of miR-153 (61). Furthermore, one of the other CircRNAs sponging miR-153, is CircPAN3, which induces chemotherapy resistance of AML through suppressing miR-153 and overexpressing XIAP (39).

LncRNAs can also regulate the expression of transcription factors related to miR-153 expression. For instance, LncRNA Taurine upregulated1 (TUG1) downregulates miR-153 indirectly in colorectal cancer through targeting KLF4, which is well-known as a miR-153 transcription factor (22). It demonstrates non-coding RNAs as molecular targets in order to discover a novel strategy for treatment of a variety of cancers.

## Some potential therapeutic drugs and suggested strategies for miR-153-related therapy

Some research have examined anti-cancerous activity of several potential therapeutic drugs and regents that are able to modulate the expression of miR-153 *in vitro* models of some cancers including glioblastoma and breast cancer. 4-phenylbutyric acid, 5-aza-2´-deoxycytidine and Olea europaea leaf extract induce apoptosis through upregulation of miR-153 targeting Bcl-2, Mcl-1 and Irs2 in glioblastoma (27, 101). Also, Mifepristone and Mifepristone derivative FZU-00,003 inhibit cell cycle progression and proliferation of breast cancer cells as well as inducing apoptosis through suppressing KLF5 expression which is mediated by overexpression of miR-153 (30, 102). Therefore, it can be suggested that regents and drugs regulating the expression of miR-153 can be extended in the experiment for a variety of tumor cells.

In addition, expanding prospective trials with larger sample sizes and further validation by independent studies on different types of tumors, miR-153 could be entered the clinical trial process and recommended as a novel treatment strategy in the future. For this purpose, there are some suggested miRNA treatment strategies including miRNA mimics and inhibitors, polymer based delivery system, nanoparticles, viral and non-viral approaches. For oncogenic miRNAs, miRNAs inhibitors, antagomiRs, anti-miR oligonucleotides, single-stranded antisense, locked nucleic acid anti-miRs, miRNA sponges prevent miRNA biogenesis or miRNA-mRNA interaction. On the other hand, for tumor suppressive miRNAs, synthetic miRNA mimics can be applied to compensate for downregulated miRNA (103).

Being unstable in the body due to numerous ribonucleases, not uptaking in the cell due to their negative charge, acting tumor microenvironment related immune cells, miRNA mimics and inhibitors have some problems with delivering in cancer cells (103). To overcome these limitations, researchers have applied several approaches to deliver therapeutic miRNA inhibitors and mimics. Using a local delivery system such as intratumoral injection of miRNA mimics or inhibitors is followed by minimal nonspecific uptake which provides effective delivery (104). Recently, some studies have been reported that local delivery of miRNAs using a polymer based delivery system, polyethylenimine and nanoparticles could achieve a significant anti-tumor effects (104–106). Mostly, local delivery systems can be applied to accessible solid tumors, thus improvement of other delivery approaches is essential to include metastatic tumors and other types of cancers. Systemic miRNA delivery systems have shown developed delivery of miRNA mimics and inhibitors (104).

In addition, using viral vectors, lentivirus, adenovirus and adeno-associated virus (AAV) have effectively delivered miRNA mimics and inhibitors. Nevertheless, the immunogenic response of viral vectors may provide serious concern in therapeutic applications (107). Therefore, improved non-viral approaches with chemical modifications is recommended for clinical applications (103). Non-viral systems are categorized into nanoparticles, lipid-based vectors (neutral/cationic), polymeric vectors (polyethylenimine, polylactic-co-glycolic acid/PLGA, chitosans, collagen and gelatin), biomaterials, and inorganic materials (gold, diamond, silica, and ferric oxide). Nanoparticles such as lipid-based systems with biocompatibility, flexibility, low immunogenicity, and versatility are the most common used approaches which constitute sphere-shaped structures composed of phospholipid bilayers in delivering exogenous nucleic acids. Furthermore, exosomes are biological nanoparticles with lipid-bilayer membrane naturally secreted from cells to mediate cell communication through intercellular transmission of nucleic acids and proteins (103, 106, 107).

## Discussion and conclusion

miR-153 is predominantly well-known as a tumor suppressive miRNA which negatively regulates genes related to cancer development. Down-regulation of miR-153 has been associated with development and progression of cancer. Expression level of miR-153 is nearly twice or more in normal tissues compared with different tumors. It can target numerous oncogenes such as AKT, CDK1, Bcl2, Mcl1, SNAI1, Snail and multiple other genes involved in proliferation, invasion, metastasis, angiogenesis and chemo/radiotherapy resistance of several types of cancer cells. Besides being as tumor suppressor, oncogenic role of miR-153 has been confirmed by some research. It can downregulate tumor suppressive genes, including PTEN, AGO1, FOXO3a and WWOX in different types of cancer. It recommends a controversial role of miR-153 as both tumor suppressor gene and oncogene which may be dependent on type and stage of cancer cells with different gene expression profiles (16–18, 88). Nevertheless, these data should be further validated by independent studies on different types and stages of tumors. On the other hand, several LncRNAs, T-UCRs and CircRNAs are introduced as oncogenic non-coding RNAs targeting and sponging miR-153 in some suggested mechanisms. Up-regulation or down-regulation of miR-153 is associated with prognostic parameters such as lymph node involvement, distant metastasis, stage and differential grade but not associated with age, gender and size of tumor in different types of cancer (**Table 4**). Ultimately, it recommends miR-153 as a potential diagnostic biomarker for detecting cancer as well as providing a novel treatment strategy to combat with several types of cancer. In conclusion, further studies, cohorts and prospective trials on a larger scale should be applied to achieve this value.

**Table 4:** Low or high expression level of miRNA-153 is associated with the prognostic parameters in different types of cancer.

MiR-153 expression	Distant metastasis	Lymph node involvement	Stage	Differential grade	Type of Cancer	References
Low	+	+	–	–	Breast cancer	(41, 43, 53, 60, 64)
	+	+	+	+	Non-small cell lung cancer	(58)
	+	+	+	+	Oral cancer	(44, 55)
	+	N.A	+	+	Melanoma	(46, 54)
	N.A	+	+	+	Esophageal squamous cell carcinoma	(47, 62)
	+	+	+	+	Pancreatic adenocarcinoma	(49)
	+	N.A	+	+	Liver cancer	(57)
	+	+	+	–	Gastric cancer	(12)
	N.A	N.A	+	+	Ovarian cancer	(36)
High	+	+	+	+	Renal cell carcinoma	(16)
	+	+	+	N.A	Prostate cancer	(17)

## Author contributions

SY, conception, providing the data and design, and manuscript writing.

## Acknowledgments

I am sincere to my colleagues at University of Isfahan for their valuable discussions.

## Conflict of interest

The author declares that the research was conducted in the absence of any commercial or financial relationships that could be construed as a potential conflict of interest.

## Publisher’s note

All claims expressed in this article are solely those of the authors and do not necessarily represent those of their affiliated organizations, or those of the publisher, the editors and the reviewers. Any product that may be evaluated in this article, or claim that may be made by its manufacturer, is not guaranteed or endorsed by the publisher.

## Glossary

**Table:**

APL	Acute promyelocytic leukemia
AAV	Adeno-associated virus
ADAM	A Disintegrin and Metalloproteinase
APP	Amyloid precursor protein
ANG1	Angiopoietin 1
AGO1	Argonaute RISC catalytic component 1
CREB	cAMP-response element binding protein
CSCs	Cancer stem cells
CITED2	Cbp/p300-interacting transactivator 2
CML	Chronic myeloid leukemia
CircRNAs	Circular RNAs
CBP	CREB-binding protein
ENCORI	Encyclopedia of RNA Interactomes
EMT	Epithelial mesenchymal transition
FOXO1	Forkhead box class O 1
FOXO3a	Forkhead box class O 3a
GPX1	Glutathione peroxidase 1
HRE	HIF1 response element
HECTD3	Homologous to the E6-associated protein carboxyl terminus domain containing 3
IGFBP-3	IGF binding protein-3
IDO1	Indoleamine 2, 3-dioxygenase 1
IGF-1	Insulin-like growth factor 1
IGF-1R	Insulin-like growth factor1 receptor
Irs-2	Insulin receptor substrate-2
JAG1	Jagged canonical notch ligand1
KIF20A	Kinesin-like protein
KLF5	Kruppel-like factor 5
LncRNAs	Long non-coding RNAs
MMP9	Matrix metalloproteinase
MTDH	metadherin
miRNA	microRNAs
mTORC2	mTOR complex 2
ncRNAs	Non-coding RNAs
NRF2	Nuclear factor erythroid 2-related factor 2
NuRD	Nucleosome remodeling and deacetylase
Pre-miRNA	Precursor miRNA
Pri-miRNAs	Primary transcripts
PKC	Protein kinase C
Rabl3	Rab-like protein 3
ROCK1	Rho associated coiled-coil containing protein kinase 1
ARHGAP18	RhoGTPase activating protein 18
RPS6KB1	Ribosomal protein S6 kinase B1
RISC	RNA-induced silencing complex
SNPs	single nucleotide polymorphisms (SNPs)
TUG1	Taurine upregulated1
TGFβR2	TGFβreceptor 2
T-UCRs	Transcribed ultra-conserved regions
VEGF	Vascular endothelial growth factor
VSMCs	Vascular smooth muscle cells
WISP1	WNT1-inducible signaling pathway protein-1
ZNRF2	Zinc and ring finger 2
ZBTB2	Zinc finger and BTB domain-containing protein 2
